# Increased expression and phosphorylation of 6-phosphofructo-2-kinase/fructose-2,6-bisphosphatase isoforms in urinary exosomes in pre-eclampsia

**DOI:** 10.1186/s12967-019-1806-6

**Published:** 2019-02-28

**Authors:** R. Ellis, M. Katerelos, S. W. Choy, N. Cook, M. Lee, K. Paizis, G. Pell, S. Walker, D. A. Power, P. F. Mount

**Affiliations:** 1 Faculty of Medicine, Dentistry and Health Sciences, University of Melbourne, Parkville, Australia; 2 Department of Nephrology, Austin Health, Studley Road, Heidelberg, Melbourne, VIC 3084 Australia; 3 Mercy Hospital for Women, Heidelberg, Australia; 4 Kidney Laboratory, Institute for Breathing and Sleep, Heidelberg, Australia

**Keywords:** Pre-eclampsia, Glycolysis, Urinary exosomes, 6-Phosphofructo-2-kinase/fructose-2, 6-Bisphosphatase, Phosphorylation

## Abstract

**Background:**

Glycolysis is altered in various kidney diseases, but little is known about glycolysis in pre-eclampsia, a multi-system disorder with major pathological effects on the kidney. Urinary exosomes provide a non-invasive alternative for studying changes in kidney metabolism. This study aims to characterise the expression and phosphorylation of isozymes of the key glycolytic regulatory protein, 6-phosphofructokinase-2-kinase/fructose-2,6-bisphosphatase (PFK-2/FBPase-2), in urinary exosomes of subjects with pre-eclampsia (PE), compared to normotensive non-pregnant (NC) and normotensive pregnant (NP) controls.

**Methods:**

A cross-sectional study of NC (n = 19), NP (n = 23) and PE (n = 29) subjects was performed. Exosomes were isolated from urine samples by differential ultracentrifugation, and then analyzed by Western blot and densitometry for expression of PFK-2/FBPase-2 isozymes (PFKFB2, PFKFB3 and PFKFB4) and phosphorylation of PFKFB2 at residues Ser483 and Ser466 and PFKFB3 at Ser461.

**Results:**

PFKFB2 expression was increased 4.7-fold in PE compared to NP (p < 0.001). PFKFB2 phosphorylation at Ser483 was increased 2.6-fold in PE compared to NP (p = 0.002). Expression of phosphorylated PFKFB2/PFKFB3 at Ser466/Ser461 was increased in PE, being present in 77.4% (95% CI 59.9–88.9%) of PE and 8.3% (95% CI 1.2–27.0%) of NP samples (p < 0.001). PFKFB3 was more commonly expressed in PE, detected in 90.3% (95% CI 74.3–97.4%) of PE and 8.3% (95% CI 1.2–27.0%) of NP samples (p < 0.001). PFKFB4 had a 7.2-fold increase in expression in PE compared to NP (p < 0.001). No significant differences between NP and NC groups were observed.

**Conclusion:**

Regulatory proteins that increase glycolysis are increased in the urinary exosomes of subjects with pre-eclampsia, suggesting that renal glycolysis may be increased in this condition.

## Background

Altered renal energy metabolism is a common characteristic of many forms of kidney disease [1–4]. In particular, changes in expression and activity of glycolytic enzymes have been demonstrated in several kidney diseases including polycystic kidney disease, ischaemic acute kidney injury and diabetic nephropathy [2, 4–6]. Pre-eclampsia is a systemic hypertensive disorder of pregnancy, which is a major cause of maternal and perinatal morbidity and mortality [7]. Renal manifestations of pre-eclampsia include proteinuria, reduced glomerular filtration rate, sodium retention and acute kidney injury [8]. Contributing factors to renal injury in pre-eclampsia include endothelial dysfunction, thrombotic microangiopathy and damage from circulating anti-angiogenic factors [9]. Notably, renal energy metabolism in pre-eclampsia is poorly understood and, specifically, renal glycolysis in pre-eclampsia has not previously been studied.

A novel method for the study of renal pathophysiology and energy metabolism is the analysis of urinary exosomes and other urinary extracellular vesicles (uEVs) [10–13]. Exosomes are extracellular microvesicles that are 40–100 nm in size and secreted by their cells of origin into the intercellular space [11]. Urinary exosomes are a subset of a larger group of secreted vesicles, which are broadly termed urinary extracellular vesicles, which also include microvesicles and apoptotic bodies [13]. Urinary exosomes and other uEVs can be used to detect in vivo changes in renal molecular expression within the pregnant population and provides a safer alternative to renal biopsy [12]. Metabolic proteins have been shown to be enriched within exosomes compared to kidney homogenates [10, 14].

A key regulator of glycolysis is 6-phosphofructo-2-kinase/fructose-2,6-bisphosphatase (PFK-2/FBPase-2). By controlling the level of fructose 2,6-bisphosphate (Fru 2,6-P2), PFK-2/FBPase-2 is the central enzyme in controlling the balance between glycolysis and gluconeogenesis [15]. This is because Fru 2,6-P2 is a powerful allosteric regulator of 6-phosphofructo-1-kinase (PFK-1) and fructose-1,6-bisphosphatase, the rate limiting enzymes in glycolysis and gluconeogenesis, respectively. PFK-2/FBPase-2 is a bifunctional enzyme, with a kinase domain at the N terminal and phosphatase domain the C terminal of the protein. The kinase domain increases levels of Fru 2,6-P2, whilst the phosphatase domain decreases the level. High levels of fructose 2,6-P2 increase PFK-1 activity and promote glycolysis, whilst inhibiting fructose-1,6-bisphosphatase. Conversely, low Fru 2,6-P2 promotes gluconeogenesis. PFK-2/FBPase-2 exists as multiple isoforms designated as PFKFB1-5, with each isoform have distinct patterns of tissue expression, activity and regulation [16]. In addition to allosteric regulation, PFK-2/FBPase-2 activity can also be regulated by phosphorylation. Phosphorylation in the C terminal regulatory domain at Ser483 and Ser466 residues of PFKFB2, and the Ser461 residue of PFKFB3, reduces the FBPase-2 activity and increases the PFK-2 activity of the enzyme, with the end result being a shift to increased glycolysis [17, 18].

This study aims to detect, characterise and compare the expression and phosphorylation of PFK-2/FBPase-2 isoforms in urinary exosomes of PE subjects, in comparison to normotensive pregnant and normotensive non-pregnant subjects. In this study we examined the specific isoforms of PFK-2/FBPase-2 reported to be expressed by the kidney, namely PFKFB2, PFKFB3 and PFKFB4 [16, 19]. The PFKFB1 (liver) isoform was not included in the study, because its expression by the kidney is reported to be absent or negligible [16, 19]. Enzymes that promote glycolysis are hypothesized to be upregulated and have increased activation in pre-eclampsia compared to normotensive pregnancy. By studying these changes in renal metabolism we can gain insight into the effects of pre-eclampsia on the kidney, informing the pathophysiology of the systemic effects of the disease, as well as help find novel biomarkers.

## Methods

### Study design and population

A non-interventional, cross-sectional study was performed across two tertiary centres, Austin Health and Mercy Hospital for Women. Three groups of subjects were included in the study, normotensive non-pregnant (NC, N = 19), normotensive pregnant (NP, N = 23) and PE (N = 29) subjects. NP and PE samples were recruited from outpatient clinics and as inpatients from The Mercy Hospital for Women and NC subjects were recruited from the University of Melbourne, Melbourne Medical School. The period of recruitment was from February 2015 to May 2018. To be involved in the study subjects had to meet the following inclusion criteria.

NC: aged greater than 18 years, no history of cardiac disease, renal disease or diabetes and not currently taking anti-hypertensive medication.

NP: aged greater than 18 years, no history of cardiac disease, renal disease or diabetes, not currently taking anti-hypertensive medication and in their second or third trimester of pregnancy.

PE: aged greater than 18 years, no pre-pregnancy history of cardiac disease, renal disease or diabetes, currently in their second or third trimester and have a diagnosis of pre-eclampsia in accordance with the Society of Obstetric Medicine of Australia and New Zealand guidelines [8].

Subjects were excluded from the study if they were age of less than 18 years or unable to provide informed consent. Specific to the NC group, subjects could not be pregnant.

Demographic and clinical data was collected for each patient from their electronic medical record of the referring institution and included: age, gestation, gravidity, parity, systolic and diastolic blood pressure, medical conditions and current medications. Prior to blood pressure measurement, subjects were required to remain seated for 5 min. Systolic and diastolic blood pressure was measured in each arm using a Welch Allyn aneroid sphygmomanometer and averaged to obtain the recorded blood pressure reading.

### Sample collection and exosome isolation

Early morning urine samples, of approximately 50 mL volume, were collected from each participant into a specimen container and immediately placed on ice. Protease inhibitor (PI) was added to the sample at a concentration of 125 µL/10 mL of urine. The sample was then centrifuged at 1600×*g*/4 °C for 10 min to remove any cellular debris. Supernatant was then removed without disturbing the pellet and divided into 10 mL tubes. These samples were then frozen and stored at − 80 °C to be processed at a later date.

Exosomes were isolated using sequential centrifugation previously described as an effective method previously for the isolation and enrichment of exosomes [10, 20]. In detail, frozen urine (10 mL) was removed from − 80 °C storage and vortexed until completely thawed. The sample was then placed into three 3 mL polycarbonate tubes, weight balanced and then centrifuged in a benchtop ultracentrifuge at 17,000×*g*/4 °C for 20 min. The supernatant from these tubes was then removed and placed into three new 3 mL polycarbonate tubes without disturbing the pellet. The new tubes were weight balanced using an analytical balance and centrifuged at 200,000×*g*/4 °C for 1 h. The supernatant was then discarded and 1 mL of phosphate buffered saline (PBS) with PI (PBS/PI) at a concentration of 125 µL/10 mL was added to the remaining pellet of each tube. The tubes were then vortexed for 30 s and all the samples pooled into one new 3 mL polycarbonate tube. The sample was weight balanced and placed into the benchtop ultracentrifuge to spin at 200,000×*g*/4 °C for 1 h. The supernatant was discarded from the tube and 30 µL of PBS/PI (125 µL/10 mL) and 7 µL of dithiothreitol (50 mg/mL) solution added to the remaining pellet to prevent aggregation of Tamm–Horsfall protein [21]. The sample was vortexed for 1 min and transferred to an Eppendorf tube and the volume adjusted to 60 μL using PBS/PI. SDS sample buffer (15 µL) was added to the sample and these exosomes were then placed in storage at − 80 °C until required for gel electrophoresis.

### Gel electrophoresis

Eppendorf tubes containing exosome samples were removed from − 80 °C storage, vortexed for 30 s and placed on a 95 °C heating block for 5 min. Aliquots (20 µL) of samples were loaded into a 10 well pre-cast polyacrylamide gel and the gel placed into a Mini-Protean 3 Cell running tank in SDS running buffer. The first well of each gel contained 20 µL of Precision Plus pre-stained protein ladder (molecular weight 10–250 kDa) to be used as a reference for protein size. The last well of each gel contained a positive control, a sample from the urine of a subject with severe features of pre-eclampsia known to highly express all proteins being measured. Samples were run under reducing conditions, with the sample buffer containing dithiothreitol at 60 mg/mL (0.39 M). The running tank was attached to a power pack and the gel run at a constant 140 V for 1 h and 10 min or until the dye in the samples reached the bottom of the gel. Gels were then placed on a polyvinylidene transfer membrane and transferred using a Transblot Turbo transfer system for 30 min at 9 V and 1 mA. The resulting membrane was then placed in a blocking solution of 5% bovine serum albumin (BSA) in tris buffered saline with tween-20 (TBST) for 30 min to 2 h on a rotary platform.

### Western blotting

After the membrane had been in blocking solution, it was then incubated in antigen specific primary antibody (in 10–15 mL of 5% BSA/TBST with 0.1% sodium azide) for either 1 h at room temperature or overnight at 4 °C on a roller mixer (Ratek). The antibodies used are listed in Table 1. The membrane was then washed for 5 min in 7.5% non-fat dry milk in TBS, followed by three 5-min washes in TBST. This was followed by a 40-min incubation in swine anti-rabbit antibody conjugated to horseradish peroxidase (Dako, Glostrup, Denmark). An enhanced chemiluminescent agent solution was then added to the membrane for 2 min. Membranes were exposed to film and developed through an automated developer (AGFA).

**Table 1:** Antibodies used in this study

Antibody target	Serial number	Antibody type	Company
PFKFB2	D7G5R	Rabbit derived monoclonal	Cell Signalling Technology Massachusetts, USA
PFKFB3	D7H4Q	Rabbit derived monoclonal	Cell Signalling Technology Massachusetts, USA
PFKFB4	ab137785	Rabbit derived polyclonal	Abcam Cambridge, UK
pSer483-PFKFB2	D4R1W	Rabbit derived monoclonal	Cell Signalling Technology Massachusetts, USA
pSer466-PFKFB2^a^	07-1539	Rabbit derived polyclonal	EMD Millipore California, USA
CD9	D801A	Rabbit derived monoclonal	Cell Signalling Technology Massachusetts, USA
ALG-2-interacting protein X (ALIX)	3A9	Mouse derived monoclonal	Cell Signalling Technology Massachusetts, USA
Tumor susceptibility gene 101 (TSG101)	T5701	Rabbit derived polyclonal	Sigma-Aldrich Missouri, USA

^a^This antibody also recognises the pSer461-PFKFB3 site

### Densitometry analysis

Films were scanned using a Canon Canoscan LiDE 20 scanner and edited using Adobe Photoshop CS3 to adjust to greyscale. ImageJ software was used to assign arbitrary numeric band density values. Normalisation of densitometry data between and within blots was performed following the normalisation by sum method [22]. CD9 is an exosomal marker used to correct for the variable exosome yield from the original urine samples [23], each band density value was divided by the corresponding CD9 expression value as a loading control for exosomal content prior to statistical analysis. Selected samples were also blotted for ALG-2-interacting protein X (ALIX) [24] and tumor susceptibility gene 101 (TSG101) [25] to confirm that our method was successfully isolating urinary exosomes.

### Statistical analysis

The unpaired t test with Welch’s correction was used to compare demographic data, due to the parametric distribution and different standard deviation values of all groups. For comparison between densitometry results, the Kruskal–Wallis with Dunn’s multiple comparisons test was applied given the non-parametric distribution of the data and the different sample sizes. For Total PFKFB2 and PFKFB4 results, samples which did not have CD9 expression were not included in the analysis. This was due to the inability to calculate the enzyme to CD9 ratio. For this same reason, any samples which did not express PFKFB2 were not included in the calculation of the PFKFB2 phosphorylated at Ser483 to total PFKFB2 ratio. To calculate differences between groups where one group had consistently absent bands, total PFKFB3 and PFKFB2/PFKFB3 phosphorylated at Ser466/Ser461, contingency tables and Fisher’s exact test were applied. Confidence intervals were calculated using the modified Wald method.

## Results

The number of participants analyzed per group were NC (N = 19), NP (N = 23) and PE (N = 29). Demographic data collected for each group is summarized in Table 2. The mean age and body mass index (BMI) of the NC group was significantly less when compared to NP (p < 0.001 for both comparisons). The differences between the NP and PE group were not statistically significant in terms of age (p = 0.30), BMI (p = 0.41) and gestation (p = 0.054). The PE group had a higher percentage of nulliparous women compared to the NP group, however this difference was not calculated to be statistically significant using Fisher’s exact test (68.97% vs. 52.2%, p = 0.26). Systolic and diastolic blood pressures were significantly higher in PE compared to NP and NC groups (p < 0.001 for both comparisons). The NP and NC groups did not show a significant difference between systolic and diastolic blood pressures (p = 0.56 and p = 0.12 respectively).

**Table 2:** Patient demographics and clinical characteristics

	NC [18]	NP [22]	PE [28]	p value		
				NC vs. NP	NC vs. PE	NP vs. PE
Age (years)	24 ± 1.7	32.7 ± 4.4	31.1 ± 6.3	< 0.001	< 0.001	0.30
BMI (kg/m^2^)	20.8 ± 1.5	27.0 ± 6.7	28.8 ± 8.5	< 0.001	< 0.001	0.41
Gestation (weeks)	N/A	32.8 ± 4.2	30.7 ± 3.0	N/A	N/A	0.054
Nulliparous (%)	N/A	52.2	69	N/A	N/A	0.26
SBP (mmHg)	108.5 ± 10.5	110.3 ± 8.7	150.5 ± 14.9	0.56	< 0.001	< 0.001
DBP (mmHg)	72.3 ± 8.1	68.7 ± 4.9	91.2 ± 9.1	0.12	< 0.001	< 0.001

Values are expressed as the mean ± the standard deviation

*SBP* systolic blood pressure, *DBP* diastolic blood pressure

p values were calculated using the unpaired t test with Welch’s correction, except for when comparing nulliparity, where Fisher’s exact test was used

### PFKFB2 expression and phosphorylation

**Fig. 1:**
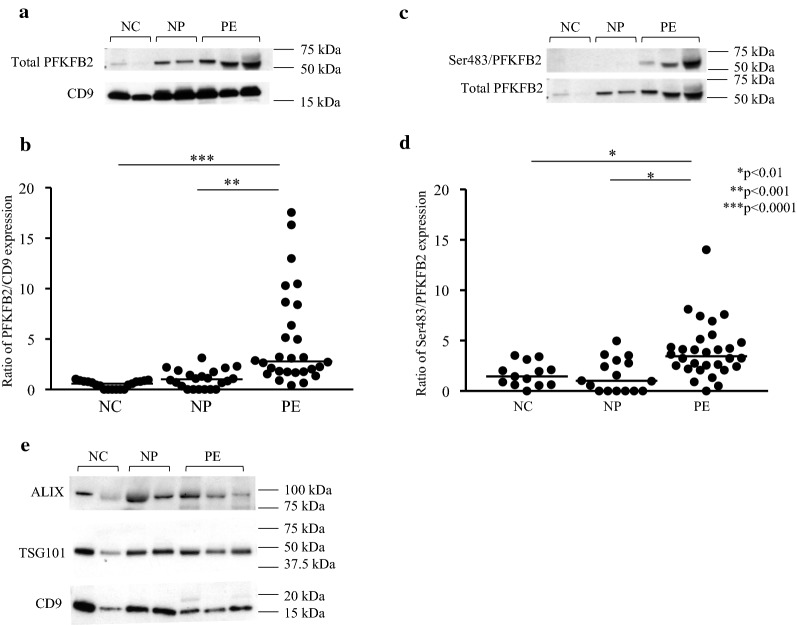
Expression of total PFKFB2 and phosphorylation of PFKFB2 at Ser483. Western blots obtained by immunoblotting antibodies directed against total PFKFB2, Ser483 phosphorylated PFKFB2 and CD9 protein (**a**, **c**). Densitometry analysis shows a 4.7-fold increase in PFKFB2 expression in the PE group as compared to the NP group, represented as a ratio of PFKFB2 to CD9 expression (**b**). **d** Shows the 2.6-fold increase in PFKFB2 phosphorylation at the Ser483 residue. Data is represented as scatter plots, with each individual patient densitometry value represented as a dot, with the horizontal line representing the median. **e** Western blot demonstrating proportional presence of the exosomal markers CD9, TSG101 and ALIX in the prepared samples

There was a 4.7-fold increase in total expression of the PFKFB2 isozyme in the PE group compared to NP (p < 0.001) (Fig. 1a, b). Total PFKFB2 expression was corrected for exosomal content, as measured by CD9 expression. Western blot for ALIX and TSG101, as confirmatory exosomal content markers, confirmed a close relationship between the levels of three independent exosomal markers (Fig. 1e). In addition to increased total expression, phosphorylation of PFKFB2-Ser483 was 2.6-fold higher in the PE group compared to NP (p = 0.025) (Fig. 1c, d). There was no difference in PFKFB2 expression and Ser483 phosphorylation between the NC and NP groups (p = 0.29 and p > 0.99 respectively).

### PFKFB3 expression

**Fig. 2:**
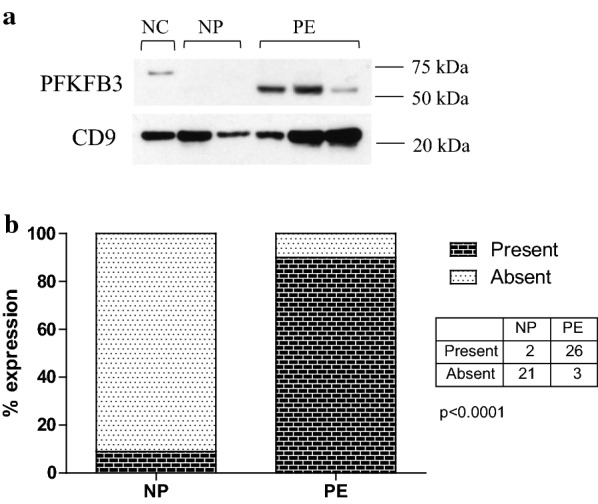
Expression of PFKFB3. Western blot obtained by immunoblotting antibodies directed against total PFKFB3 protein (**a**). The positive control lane used a sample from the urine of a subject with severe features of pre-eclampsia known to highly express all proteins being measured. **b** Shows the percentage of samples which had detectable bands on Western blot analysis, with the table showing the actual number of patients. PFKFB3 was more commonly expressed in PE, detected in 90.3% (95% CI 74.3–97.4%) of PE and 8.3% (95% CI 1.2–27.0%) of NP samples (p < 0.001)

PFKFB3 was undetectable in most NP participants, hence PFKFB3 expression was analyzed by contingency tables (present vs. absent) rather than by densitometry. PFKFB3 was more commonly expressed in PE compared to NP, detected in 90.3% (95% CI 74.3–97.4%) of PE and only 8.3% (95% CI 1.2–27.0%) of NP samples (Fig. 2a, b) (p < 0.001). There was no difference in PFKFB3 expression between the NC and NP groups (p = 0.50). Within the NC group, PFKFB3 was not expressed in any of the samples analyzed, although a non-specific band at a higher molecular weight was observed in one lane (Fig. 2a). The calculated MW of the non-specific band was 63 kDa, compared to 54 kDa for PFKFB3.

### PFKFB2/PFKFB3 phosphorylation at Ser466/Ser461

**Fig. 3:**
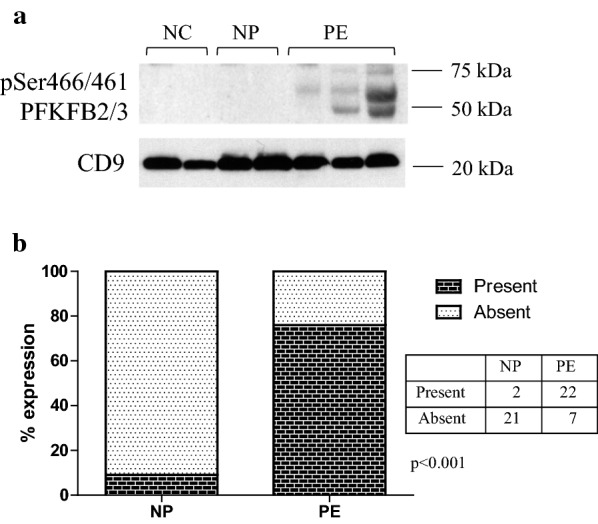
Phosphorylation of PFKFB2/PFKFB3 at Ser466/Ser461. **a** shows Western blot obtained by immunoblotting antibody directed against Ser466/Ser461 on PFKFB2/PFKFB3. The positive control lane used a sample from the urine of a subject with severe features of pre-eclampsia known to highly express all proteins being measured. **b** Shows the percentage of samples which had detectable bands on Western blot analysis, with the table showing the actual number of patients. Phosphorylated PFKFB2/PFKFB3 was detected in 77.4% (95% CI 59.9–88.9%) of PE and 8.3% (95% CI 1.2–27.0%) of NP samples (p < 0.001)

We have found that the EMD Millipore antibody against the PFKFB2-Ser466 phospho-site, also recognizes the homologous PFKFB3-Ser461 phospho-site. This cross reactivity was first identified in animal tissues in studies of genetically modified mice, and confirmed by immunoprecipitation studies (data not shown). In retrospect this was unsurprising given the strong sequence homology of these two phospho-sites (PFKFB2-S466, MRRN**S**FTPLSSS: PFKFB3-S461, MRRN**S**VTPLASP). As with PFKFB3 expression, bands were consistently absent in most NC and NP participants, so analysis was by contingency tables (present vs. absent) rather than by densitometry. Ser466/461 phosphorylated PFKFB2/3 expression was increased in PE compared to NP, with bands on Western blots present in 77.4% (95% CI 59.9–88.9%) of PE samples and only 8.3% (95% CI 1.2–27.0%) of NP samples (Fig. 3a, b) (p < 0.001). There was no difference in Ser466/Ser461 phosphorylation levels between NP and NC groups (p = 0.49), with no Ser466/Ser461 phosphorylation detected in any of the NC samples. Figure 3 shows this data represented as a contingency table. Of the two patients in the NP group positive for PFKFB2/PFKFB3 phosphorylation, one of these was also one of the two patients who was positive for PFKFB3 expression. There was also one other patient positive for PFKFB2/PFKFB3 phosphorylation but negative for total PFKFB3.

### PFKFB4 expression

**Fig. 4:**
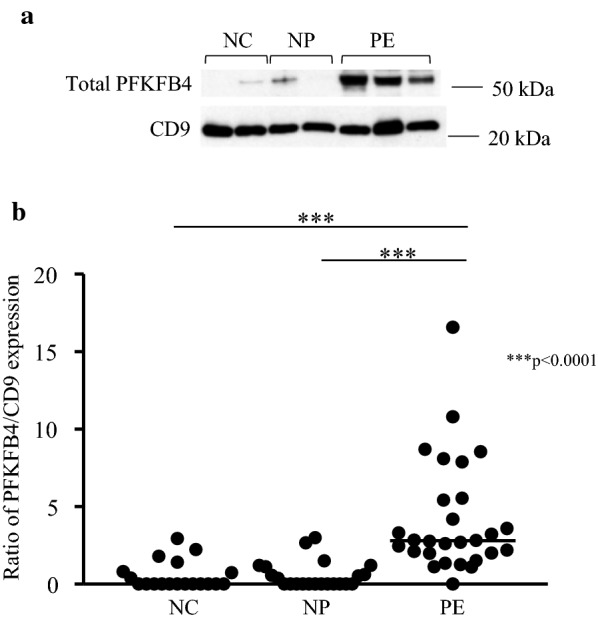
Expression of PFKFB4. **a** Shows Western blots obtained by immunoblotting antibodies directed against total PFKFB4 and CD9 protein. **b** Shows the 7.2-fold increase in PFKFB4 expression, represented as a ratio of PFKFB4 to CD9 levels. Data is represented in as a scatter plot, with each individual patient densitometry value represented as a dot, with the horizontal line representing the median

This isozyme demonstrated the largest increase in levels, with a 7.2-fold increase in expression of PFKFB4 in PE compared to NP (p < 0.001) (Fig. 4a, b). There was no significant difference in PFKFB4 expression between the NC and NP groups (p > 0.99).

## Discussion

**Fig. 5:**
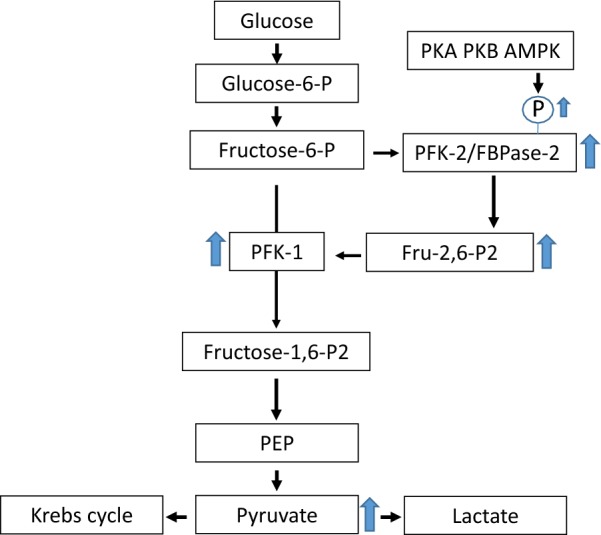
Glycolysis pathway showing how increased PFK-2/FBPase-2 expression and phosphorylation, stimulates glycolysis via increased fructose-2,6-bisphosphate synthesis acting as a powerful allosteric activator of PFK-1. The arrows show how the changes observed in PE are predicted to increase renal glycolysis. *PFK-1* phosphofructokinase 1, *PFK-2/FBPase-2* 6-phosphofructokinase-2-kinase/fructose-2,6-bisphosphatase, *Fru 2,6-P2* fructose 2,6-bisphosphate, *PEP* phosphoenolpyruvate, *AMPK* AMP-activated protein kinase, *PKA* protein kinase A, *PKB* protein kinase B

The principal finding of this study is enzymes that promote the forward reaction of glycolysis, PFKFB2-4, have increased expression and phosphorylation, as detected within urinary exosomes, in subjects with pre-eclampsia when compared to normotensive pregnant and normotensive non-pregnant subjects. This suggest that glycolysis may be increased in the kidneys of women with pre-eclampsia (Fig. 5).

PFKFB2, considered to be the dominant PFK-2/FBPase-2 isoform found within the kidney [16], was found to have increased levels in pre-eclampsia in this study. There is limited data on the role this enzyme plays in kidney disease. Muller et al. [26] found that when PFKFB2 is expressed in lower than average levels in the kidney, this correlates with worsening diabetic nephropathy. But it cannot be determined from this study, however, whether downregulation of PFKFB2 has a direct detrimental effect on the kidney. In the Muller et al. study, the allele on the PFKFB2 gene which contributes to this downregulation is also linked with higher BMI and lower insulin secretion, which could also introduce significant confounding variables. Further research is required to determine whether PFKFB2 upregulation is protective or maladaptive in renal injury. Therefore, whilst upregulation of PFKFB2 in urinary exosomes in pre-eclampsia predicts increased glycolysis, further studies are required to determine whether this effect is detrimental or adaptive.

PFKFB3, previously known as inducible PFK-2, was also observed in this study to be upregulated in pre-eclampsia. One of the major stimuli for PFKFB3 expression is hypoxia, acting through hypoxia-inducible factor 1α (HIF-1α), which binds to the PFKFB3 gene promoter region [27, 28]. Interestingly, HIF-1α is increased in the plasma of women with pre-eclampsia [29], raising the possibility that this might explain the increase in PFKFB3 expression with pre-eclampsia that was observed in the present study. Furthermore, a study by Lan et al. [4] that found that HIF-1α, PFKFB3, and other glycolytic enzymes were increased in expression following renal ischaemia–reperfusion injury in a rat model. Kidneys in pre-eclampsia are likely to be subjected to ischaemia, secondary to injury mechanisms including increased circulating anti-angiogenic factors, endothelial dysfunction and thrombotic microangiopathy. Hence, whilst various mechanisms might contribute to the increased PFKFB3 expression observed in pre-eclamptic subjects, it is potentially through a hypoxia or ischemia induced pathway. Notably, PFKFB3 has the highest kinase:phosphatase activity ratio of the various PFK-2/FBPase-2 isozymes [30, 31], indicating that the increase in PFKFB3 observed in the present study, is predicted to substantially increase Fru 2,6-P2 synthesis, leading to increased glycolysis.

We also observed increased expression of PFKFB4 in the pre-eclampsia group of this study. Despite the fact that little is known yet about the specific role of PFKFB4 in the kidney, we included it in this study on the basis of reports that this isoform is detectable in the kidney by immunohistochemistry [16, 19]. We found that PFKFB4 was detectable in urinary exosomes in control subjects, and considerably increased in pre-eclampsia subjects. Similarly to PFKFB3, expression of PFKFB4 is reported to be increased under hypoxic conditions via HIF-1α [32]. This suggests that there could be similar mechanisms to explain the increases in PFKFB4 and PFKFB3, although future studies will be needed to address this.

It is likely that the changes observed in this study correlate to increased renal glycolysis. Despite that isozymes PFKFB2-4 all have bifunctional kinase and phosphatase activity, in each case the kinase activity is predominant, hence promoting glycolysis. For example, the reported kinase:phosphatase ratios for PFKFB2, PFKFB3 and PFKFB4 are 1.8:1, 107:1 and 4.7:1 respectively [30]. PFKFB2 has the lowest ratio of the three enzymes, however in this study phosphorylation of Ser483 and Ser466 was measured. Phosphorylation at these sites increases kinase activity and the rate of glycolysis, and therefore the observation of increased PFKFB2 expression and phosphorylation in urinary exosomes in pre-eclampsia predicts increased glycolytic activity. Phosphorylation of the Ser466 sites on PFKFB2 occurs through AMP-activated protein kinase, an enzyme activated by energy stress, such as during hypoxic conditions [33]. However, it is not known whether AMPK is activated in the kidney during pre-eclampsia. On PFKFB2, Ser466 and Ser483 can also be phosphorylated by protein kinase A and B [15, 33, 34].

When interpreting the results of PFKFB2 phosphorylated at Ser466, it needs to be considered that the primary antibody used was also detecting Ser461 phosphorylation on PFKFB3. The amino acid sequence surrounding these phospho-sites are almost identical [18] which results in cross-reactivity of the primary antibody used to detect Ser466. However, phosphorylation at both of these sites leads to an increase in Fru 2,6-P2 levels and therefore detecting increased activation of either PFKFB2 or PFKFB3 at Ser 466 or Ser461 indicates stimulated glycolysis irrespective of the actual protein being identified [18].

In normal physiology, the kidney meets its high metabolic demand from various fuel sources including fatty acids, ketones, triglycerides and glucose [35], with the predominant metabolic source being oxidation of fatty acids. In certain scenarios, increased renal glycolysis appears to be harmful. For example, a metabolic shift towards glycolysis has been associated with proximal tubular atrophy following acute kidney injury [4]. Furthermore, enhanced renal glycolysis is implicated in the progression of autosomal dominant polycystic kidney disease [36], and inhibition of aerobic glycolysis is reported to slow progression of this condition [5]. It is not established, however, that increased renal glycolysis is always harmful. A consideration is that in pre-eclampsia the energy consuming process of sodium reabsorption is increased [12, 37], raising the possibility that increased renal glycolysis in PE may reflect an adaptive mechanism to meet the increased requirement for ATP synthesis. One situation in which increased renal glycolysis appears protective is diabetic nephropathy, in which activation of pyruvate kinase M2 is observed to protect against disease progression and mitochondrial dysfunction [2]. Overall, further studies are needed to understand whether an increase in renal glycolysis with PE is harmful or adaptive.

A limitation of our study is its cross-sectional nature, such that the patients in the PE group already had established PE diagnosis at the time of the urine collection. Further studies will be required to determine the time course of the appearance of PFK-2/FBPase-2 isoforms in the urine of women with PE. This will be important in determining whether urine analysis of PFK-2/FBPase-2 isoforms could be useful as a diagnostic biomarker for PE. Time course studies may also provide insight into whether increased urinary excretion of PFK-2/FBPase-2 isoforms is likely to be a cause or a consequence of PE. Another unknown is whether increased PFK-2/FBPase-2 isoforms in urinary exosomes is specific to PE, or may also occur in other forms of kidney disease. Further studies will be required to address this. It is also not currently known if PFK-2/FBPase-2 isoforms exist in a soluble form in urine from women with preeclampsia. Historically, diagnosis of PE has relied on the detection of end organ consequences, most commonly proteinuria [8]. The ideal biomarker for early diagnosis of pre-eclampsia would identify the condition in the first trimester, before clinical manifestations, and when the administration of therapies such as aspirin are most likely to be effective [38]. A wide variety of serum markers and imaging methods have been studied for this purpose but none are yet to achieve clinical application [38]. A potential advantage of urinary biomarkers and the isolation of uEVs is their potential to non-invasively provide a direct and early read-out of renal pathophysiological changes.

Another consideration in the interpretation of our study is the potential that in addition to exosomes our samples may include other uEVs. The presence of exosomes in our samples is supported by the expression of the exosomal markers CD9, TSG101 and ALIX, and the fact that we used a sequential ultracentrifugation methods reported to be effective for the isolation of exosomes from biological fluids [10, 20]. Nonetheless, it is acknowledged that there is currently no consensus on optimal methods to isolate and purify extracellular vesicles [25], we cannot exclude the possibility that our preparation could include some contaminations by other vesicles such as microvesicles and apoptotic bodies. It is also possible that the pattern of uEVs is altered in PE. Another limitation is that the cellular origin of the PFK-2/FBPase-2 isoforms detected in urinary exosomes in the present study is unclear. It has previously been shown that in PE uEVs are detectable from both tubular [12] and podocyte [39] origins.

## Conclusion

In conclusion, glycolytic proteins, PFKFB2-4, are upregulated in urinary exosomes isolated from pre-eclamptic subjects, suggesting that renal glycolysis may be increased in pre-eclampsia. Furthermore, phosphorylation of PFKFB2 and PFKFB3 is increased in pre-eclampsia, providing further evidence for a metabolic shift to increased glycolysis. Whether this is a maladaptive response, as evident in other kidney diseases, remains unknown. Given that these metabolic enzymes are consistently upregulated in urinary exosomes of pre-eclamptic subjects, these isozymes may also have a role as potential biomarkers of the disease.

## Abbreviations

PFK-2/FBPase-2: 6-phosphofructokinase-2-kinase/fructose-2,6-bisphosphatase
PFKFB1-4: 6-phosphofructokinase-2 kinase/fructose-2,6-bisphosphatase isoforms 1-4
Fru 2,6-P2: fructose 2,6-bisphosphate
PFK-1: phosphofructokinase 1
HIF1α: hypoxia-inducible factor 1α
